# Barriers and facilitators related to healthcare practitioner use of real time prescription monitoring tools in Australia

**DOI:** 10.3389/fpubh.2023.1175791

**Published:** 2023-09-12

**Authors:** Dimi Hoppe, Chaojie Liu, Hanan Khalil

**Affiliations:** School of Psychology and Public Health, La Trobe University, Melbourne, VIC, Australia

**Keywords:** real time prescription monitoring, prescription drug monitoring programs, healthcare, pharmacists, prescribers, barriers

## Abstract

**Introduction:**

Real time prescription monitoring tools have been implemented in Australia to address the growing concerns of drug misuse, drug-related mortality and morbidity. The objective of this pilot study is to investigate the barriers and facilitators related to healthcare practitioner use of real time prescription monitoring tools.

**Methods:**

An online survey was distributed to Australian prescribers and pharmacists who use a real time prescription monitoring tool. Data analysis included descriptive statistics, chi-square tests and multivariate logistic regression analyses.

**Results:**

A total of 102 questionnaires were analyzed. Practitioners mainly agreed that the tool was easy to use (*n* = 64; 66.7%) and access (*n* = 56; 57.7%), and the data was easy to interpret (*n* = 77; 79.4%). Over half agreed that they wanted training to guide clinical actions (*n* = 52; 55.9%) and clinical guidelines or guidance on what to do with the RTPM findings (*n* = 51; 54.8%). Prescribers were more likely to report difficulties with workplace access to a computer or the internet (*n* = 7; 21.2%) compared with pharmacists (*n =* 6; 9.2%; *p =* 0.037). Practitioners working in community settings (*n* = 59; 57.9%; *p* = 0.022) and those with 1–10 years practice experience (*n* = 45; 44.2%; *p* = 0.036) were more likely to want training to guide clinical actions in response to RTPM information.

**Conclusion:**

This is the first known study to investigate the barriers and facilitators related to practitioner use of RTPM tools in Australia. The results from this study can inform further research to gain an understanding of healthcare practitioners use of RTPM tools, and how to minimize barriers and optimize use for the essential delivery of quality healthcare.

## 1. Introduction

In 2020, 61 million people used opioids for non-medical reasons (1.2% of the global population) (1). Opioids were associated with the highest mortality rates present in 75% of fatal overdoses in the United States in 2020 and in 76% of European Union cases in 2019 (1). Overdose deaths in Australia have exceeded the road toll for seven consecutive years and in 2020, pharmaceutical opioids were involved in 47.3% of fatalities (2).

Internationally, a range of harm minimization policies, strategies and interventions are employed, with the understanding that multiple initiatives must be implemented to successfully combat drug misuse and drug-related mortality and morbidity (1). Evidence-based interventions include naloxone to reverse the effects of opioid overdose, drug-related treatment for substance use disorders, needle-syringe programs, and psychosocial and rehabilitation therapies (1, 3–5). In addition, electronic databases that monitor prescribing and dispensing of controlled substances including opioids have been widely implemented in the United States, some places in Europe, and more recently in Australia (6). The implementation of these databases, known in the US as prescription drug monitoring programs (PDMPs) and in Australia as real time prescription drug monitoring (RTPM) tools require a substantial degree of technological sophistication to operate efficiently and effectively (7). The evolution of PDMPs from conception to date, and the implementation of information systems in the Australian healthcare sector such as My Health Record, telehealth, and electronic prescriptions clearly demonstrates the expansion of digital health infrastructure across the globe, with the intention of improving access to clinical information and the delivery of quality healthcare (3, 8, 9).

Maximizing the value of information technologies in healthcare is not without its challenges. Firstly, these systems must be developed and implemented so they seamlessly integrate with existing healthcare programs. Secondly, they must support healthcare practice and comply with legal and ethical obligations. Finally, professional practice guidelines must be updated to incorporate digital health systems, and tailored education provided to healthcare practitioners who are expected to use these tools in practice (3). Successful uptake of clinical decision support systems such as RTPM tools depends on effective execution of this process, as well as other factors including clinicians’ perceived benefit to patients (10, 11). The overarching purpose of RTPM tools is to improve patient health outcomes by facilitating safer clinical decisions and reducing harm (10, 12). These outcomes rely on healthcare practitioners effectively using RTPM tools. It is widely accepted that practitioner RTPM tool utility and barriers to use play an important part in whether the tools will achieve their desired goals. Previous research has investigated barriers and facilitators to PDMP use by healthcare practitioners in the United States, however in Australia the literature is limited (9, 12–19).

The objective of this study is to investigate the barriers and facilitators related to healthcare practitioner use of real time prescription monitoring tools. The study addresses the following research questions:

1. What are the barriers and facilitators (including training and education) associated with the use of RTPM tools?
2. What do prescribers and pharmacists perceive will make RTPM tools more useful in clinical practice?

## 2. Methods

### 2.1. Design and sample

This pilot study was part of a larger research project that used a survey questionnaire to evaluate healthcare practitioner use of RTPM tools including practice perspectives and RTPM-informed clinical interventions. The online survey was developed using REDCap 12.4.8 (Vanderbilt, Tennessee, United States, August 2022) and informed by previously published studies that examined similar themes (16, 18). Nine healthcare practitioners reviewed the questionnaire to assess clarity and to test face validity. The survey instrument questions are included in Supplementary material. The survey questions reported in this study collected demographic information and examined healthcare practitioner barriers and facilitators to use. Open-ended questions investigated practitioner recommendations for RTPM tool improvement and invited any further comments not covered in the survey. Results on clinical practice perspective and RTPM-informed clinical interventions are reported elsewhere (20).

The study population includes Australian Health Practitioner Regulation Authority (AHPRA) registered prescribers and pharmacists who are authorized to access and use their state or territory RTPM tool. Prescribers and pharmacists who were not using a RTPM tool were excluded from the study.

### 2.2. Recruitment

Between December 2021 and February 2022, key stakeholders with healthcare practitioner membership such as professional practice organizations and primary health networks were invited to distribute the survey to their members. A survey link, QR code, information sheet and consent form were provided. The study researcher, also a practicing pharmacist, declared their dual roles in the study information. Participation was voluntary and responses were deidentified.

A total of 267 potential participants accessed the survey. Removal of incomplete data and practitioners not registered to use or never used a RTPM tool resulted in 102 questionnaires for analysis. This sample size allows estimation of a proportion of greater than 50% at a relative error range of less than 10% and up to 10 predictors in regression modeling (21).

### 2.3. Data analysis

Data were analyzed using Statistical Package for Social Sciences (SPSS Version 28.0.1.1. IBM Corp). Descriptive statistics were used to summarize participant characteristics and responses. Chi-square test was conducted to examine the differences between prescribers and pharmacists and perceived tool use.

Multivariate logistic regression analyses were performed to examine participant characteristics as predictors of RTPM-related barriers and facilitators. Content analysis was used to analyze responses to the open-ended questions.

### 2.4. Ethics statement

The study was approved by La Trobe University Human Research Ethics Committee, Australia (HEC21259).

## 3. Results

### 3.1. Sample characteristics

The mean age of respondents (*n =* 102) was 43.3 years and 65.7% (*n* = 67) identified as female. The main healthcare practitioner type was pharmacist (*n =* 67; 65.7%), followed by medical practitioner (*n =* 34; 33.4%). More than half of the respondents practiced in community settings (*n =* 59; 57.9%). Over half the respondents use a RTPM tool more than 3 times on a typical workday (*n =* 56; 54.9%). Twenty-two respondents stated they were not registered to use a RTPM tool. Reported reasons for non-registration were not knowing they had to or how to register, not having access to a RTPM tool at their workplace, and because use is not mandatory in their state/territory. Six respondents who were registered to use the tool reported that they had never used it. Reasons for never having used a RTPM tool included that it was only recently implemented in their practice, or they were not prescribing monitored medicines.

Table 1 outlines participant demographics and characteristics.

**Table 1:** Healthcare practitioner demographics and characteristics (*n* = 102).

Characteristic	Frequency *n* (%)	Characteristic	Frequency *n* (%)
*Gender*			
Female	67 (65.7%)	*Main practice—geographic location (state/territory)*	
Male	33 (32.4%)	Victoria	44 (43.2%)
Other (prefer not to answer, non-binary)	2 (1.9%)	Queensland	29 (28.5%)
		South Australia	18 (17.6%)
*Age (years)*		New South Wales	5 (4.9%)
20–29	24 (23.5%)	Australian Capital Territory	3 (2.9%)
30–39	23 (22.5%)	Tasmania	3 (2.9%)
40–49	17 (16.7%)		
50–59	20 (19.6%)		
60+	18 (17.7%)	*Main practice—geographic region*	
Mean age (SD)	43.3 (14.8)	Metro	68 (66.7%)
		Regional	27 (26.4%)
*Healthcare practitioner type*		Rural	7 (6.9%)
Pharmacist	67 (65.7%)		
Medical practitioner	34 (33.4%)	*Practice experience (years)*	
Other prescribers (nurse practitioner)	1 (0.9%)	<1	5 (4.9%)
		1–10	45 (44.2%)
		11–20	15 (14.7%)
*Practice setting*		>20	36 (35.3%)
Community	59 (57.9%)	Other (not reported)	1 (0.9%)
General practice	21 (20.7%)		
Hospital	13 (12.7%)	*Hours of practice (in an average week)*	
Emergency department	5 (4.9%)	<10	10 (9.8%)
Private clinic	3 (2.9%)	11–20	12 (11.7%)
Other (medicines regulation)	1 (0.9%)	>20	80 (78.5%)
*Prescriber specialties*			
Primary healthcare	22 (64.7%)		
Emergency medicine	3 (8.9%)	*Pharmacist specialties*	
Hospital	3 (8.9%)	Primary healthcare (community)	58 (86.6%)
Anesthesiology	2 (5.9%)	Hospital	7 (10.4%)
Addiction medicine	1 (2.9%)	Emergency medicine	1 (1.5%)
Psychiatry	1 (2.9%)	Primary healthcare (general practice)	1 (1.5%)
Sport and exercise	1 (2.9%)		
Dentistry	1 (2.9%)		
*RTPM tool use (on a typical day)*		*Mean = 2.75 (SD = 1.07)*	
<1 time per day		14 (13.7%)	
1–2 times per day		32 (31.4%)	
3–5 times per day		21 (20.6%)	
>5 times per day		35 (34.3%)	

### 3.2. Barriers and facilitators to RTPM tool use

Table 2 outlines responses to statements associated with barriers and facilitators to RTPM tool use. Access to hardware and software was not a barrier to RTPM tool use according to the majority of participants who disagreed with the statements that they had limited or no access to a computer or the internet (*n =* 85; 86.8%) or to the RTPM tool at their workplace (*n =* 82; 83.7%). In addition, practitioners mainly agreed that the tool was easy to use (*n =* 64; 66.7%) and access (*n =* 56; 57.7%), and the data was easy to interpret (*n =* 77; 79.4%). Less than half of the respondents agreed that the tool was integrated into their workflow process (*n =* 45; 46.4%) and workflow integration was identified as a potential barrier with 59.8% of respondents (*n =* 58) disagreeing with the statement that using the RTPM tool does not disrupt workflow. Practitioner response was divided on the statement related to time constraints, 37.1% (*n =* 36) agreed that they had sufficient time to use the RTPM tool and 41.3% (*n =* 40) disagreed. The majority of respondents appeared to be in favor of mandatory use (*n =* 70; 72.2%). Only 13.4% (*n =* 13) agreed that tool use should be voluntary. Chi-square test revealed no statistically significant differences between pharmacists and prescribers and perceptions of barriers and facilitators to tool use. The multivariate regression analyses showed no significant differences between the pharmacists and the prescribers in perceived barriers and facilitators after adjustment for variations of the sociodemographic and job factors, except that prescribers were more likely to report difficulties in IT access (*n* = 7; 21.2%; *p =* 0.037).

**Table 2:** Barriers and facilitators to RTPM tool use.

Item	*Total*	*Pharmacists*	*Prescribers*	*p*-value^*^
	*n* (%)	*n* (%)	*n* (%)	
*I have limited or no access to a computer or internet at my workplace*				0.120
Agree	3 (3.0%)	2 (3.1%)	1 (3.0%)	
Disagree	85 (86.8%)	59 (90.8%)	26 (78.8%)	
Neutral	10 (10.2%)	4 (6.1%)	6 (18.2%)	
M (SD)	4.55 (0.84)	4.58 (0.80)	4.48 (0.90)	
*I have limited or no access to the RTPM tool at my workplace*				0.131
Agree	7 (7.1%)	4 (6.1%)	3 (9.0%)	
Disagree	82 (83.7%)	57 (87.8%)	25 (75.8%)	
Neutral	9 (9.2%)	4 (6.1%)	5 (15.2%)	
M (SD)	4.38 (0.99)	4.47 (0.92)	4.21 (1.11)	
*The RTPM tool is easy to use*				0.526
Agree	64 (66.7%)	43 (68.3%)	21 (63.6%)	
Disagree	21 (21.9%)	15 (23.8%)	6 (18.2%)	
Neutral	11 (11.4%)	5 (7.9%)	6 (18.2%)	
M (SD)	2.46 (1.16)	2.46 (1.18)	2.48 (1.14)	
*The RTPM tool is easy to access*				0.180
Agree	56 (57.7%)	37 (57.8%)	19 (57.6%)	
Disagree	29 (29.9%)	22 (34.4%)	7 (21.2%)	
Neutral	12 (12.4%)	5 (7.8%)	7 (21.2%)	
M (SD)	2.67 (1.19)	2.70 (1.23)	2.60 (1.14)	
*I have sufficient time to use the RTPM tool*				0.116
Agree	36 (37.1%)	23 (35.9%)	13 (39.4%)	
Disagree	40 (41.3%)	30 (46.9%)	10 (30.3%)	
Neutral	21 (21.6%)	11 (17.2%)	10 (30.3%)	
M (SD)	3.17 (1.29)	3.28 (1.29)	2.96 (1.28)	
*The RTPM tool does not disrupt workflow*				0.749
Agree	24 (24.7%)	16 (25.0%)	8 (24.2%)	
Disagree	58 (59.8%)	39 (60.9%)	19 (57.6%)	
Neutral	15 (15.5%)	9 (14.1%)	6 (18.2%)	
M (SD)	3.53 (1.19)	3.59 (1.23)	3.42 (1.14)	
RTPM data is easy to interpret				0.054
Agree	77 (79.4%)	49 (76.6%)	28 (84.8%)	
Disagree	12 (12.4%)	11 (17.2%)	1 (3.1%)	
Neutral	8 (8.2%)	4 (6.2%)	4 (12.1%)	
M (SD)	2.2 (0.88)	2.29 (0.98)	2.03 (0.63)	
My workplace has a policy that supports RTPM tool use				0.903
Agree	52 (53.6%)	37 (57.8%)	15 (45.5%)	
Disagree	17 (17.5%)	11 (17.2%)	6 (18.1%)	
Neutral	28 (28.9%)	16 (25.0%)	12 (36.4%)	
M (SD)	2.43 (1.15)	2.37 (1.14)	2.54 (1.17)	
I am confident in my response to suspected misuse				0.417
Agree	70 (72.2%)	49 (76.6%)	21 (63.6%)	
Disagree	7 (7.2%)	6 (9.3%)	1 (3.1%)	
Neutral	20 (20.6%)	9 (14.1%)	11 (33.3%)	
M (SD)	2.2 (0.88)	2.21 (0.93)	2.18 (0.80)	
I have sufficient resources to act on RTPM information				0.327
Agree	61 (64.2%)	39 (61.9%)	22 (68.7%)	
Disagree	12 (12.7%)	10 (15.9%)	2 (6.3%)	
Neutral	22 (23.1%)	14 (22.2%)	8 (25.0%)	
M (SD)	2.42 (0.97)	2.50 (1.01)	2.25 (0.87)	
The RTPM tool is integrated into my workflow process				0.686
Agree	45 (46.4%)	31 (48.4%)	14 (42.4%)	
Disagree	32 (33.0%)	22 (34.4%)	10 (30.3%)	
Neutral	20 (20.6%)	11 (17.2%)	9 (27.3%)	
M (SD)	2.82 (1.25)	2.84 (1.23)	2.78 (1.31)	
I am concerned about patient privacy or data security when using the RTPM tool				0.802
Agree	14 (14.4%)	9 (14.1%)	5 (15.2%)	
Disagree	66 (68.1%)	43 (67.1%)	23 (69.6%)	
Neutral	17 (17.5%)	12 (18.8%)	5 (15.2%)	
M (SD)	3.71 (1.02)	3.67 (0.99)	3.78 (1.08)	
RTPM tool use should be voluntary				0.758
Agree	13 (13.4%)	8 (12.5%)	5 (15.2%)	
Disagree	70 (72.2%)	48 (75.0%)	22 (66.6%)	
Neutral	14 (14.4%)	8 (12.5%)	6 (18.2%)	
M (SD)	3.93 (1.13)	4.03 (1.11)	3.75 (1.17)	
I am concerned about the legal ramifications of using the RTPM tool				0.482
Agree	23 (23.7%)	17 (26.6%)	6 (18.2%)	
Disagree	54 (55.7%)	34 (53.1%)	20 (60.6%)	
Neutral	20 (20.6%)	13 (20.3%)	7 (21.2%)	
M (SD)	3.45 (1.11)	3.37 (1.13)	3.60 (1.08)	
RTPM tool use takes time away from patient care				0.792
Agree	22 (22.7%)	14 (21.9%)	8 (24.2%)	
Disagree	57 (58.8%)	42 (65.6%)	15 (45.5%)	
Neutral	18 (18.5%)	8 (12.5%)	10 (30.3%)	
M (SD)	3.44 (1.19)	3.59 (1.21)	3.15 (1.12)	
RTPM tool use should be reimbursed				0.362
Agree	48 (49.5%)	38 (59.4%)	10 (30.3%)	
Disagree	24 (24.7%)	14 (21.8%)	10 (30.3%)	
Neutral	25 (25.8%)	12 (18.8%)	13 (39.4%)	
M (SD)	2.59 (1.19)	2.42 (1.21)	2.93 (1.08)	

* *p*-value of Chi-square or Fisher’s Exact Test (agree vs. disagree—neutral positioned according to question).

### 3.3. Training and resources related to RTPM tool use

Practitioner responses related to RTPM tool training and resources were mainly evenly distributed (Table 3). More respondents agreed than disagreed that they had received sufficient training (*n =* 45; 48.4%) and resources (*n =* 55; 59.1%) on how to use the tool. Over half agreed that they wanted training to guide clinical actions (*n =* 52; 55.9%) and clinical guidelines or guidance on what to do with the RTPM findings (*n =* 51; 54.8%). Chi-square test revealed no statistically significant differences between pharmacists and prescribers and perceptions of training and resources related to tool use. The multivariate logistic regression models indicated that practitioners working in community settings (*n* = 59; 57.9%; *p* = 0.022) and those with 1–10 years practice experience (*n* = 45; 44.2%; *p* = 0.036) were more likely to want training to guide clinical actions in response to RTPM information.

**Table 3:** Training and resources related to RTPM tool use.

Item	*Total*	*Pharmacists*	*Prescribers*	*p*-value^*^
	*n* (%)	*n* (%)	*n* (%)	
*I received sufficient training on how to use the RTPM tool*				0.638
Agree	45 (48.4%)	30 (47.6%)	15 (50.0%)	
Disagree	31 (33.3%)	20 (31.8%)	11 (36.7%)	
Neutral	17 (18.3%)	13 (20.6%)	4 (13.3%)	
M (SD)	2.84 (1.23)	2.82 (1.18)	2.90 (1.34)	
*I have access to sufficient resources on how to use the RTPM tool*				0.651
Agree	55 (59.1%)	39 (61.9%)	16 (53.3%)	
Disagree	18 (19.4%)	13 (20.6%)	5 (16.7%)	
Neutral	20 (21.5%)	11 (17.5%)	9 (30.0%)	
M (SD)	2.50 (1.04)	2.49 (1.07)	2.53 (1.00)	
*I have access to sufficient RTPM tool support (technology, peer, practice support)*				0.797
Agree	42 (45.6%)	27 (43.5%)	15 (50.0%)	
Disagree	23 (25.1%)	15 (24.2%)	8 (26.7%)	
Neutral	27 (29.3%)	20 (32.3%)	7 (23.3%)	
M (SD)	2.76 (1.07)	2.77 (1.06)	2.73 (1.11)	
*I want training on how to interpret RTPM data*				0.161
Agree	25 (26.9%)	21 (33.3%)	4 (13.3%)	
Disagree	46 (49.5%)	28 (44.5%)	18 (60.0%)	
Neutral	22 (23.6%)	14 (22.2%)	8 (26.7%)	
M (SD)	3.29 (1.06)	3.17 (1.07)	3.53 (1.04)	
*I want training to guide clinical actions I can take (as a result of RTPM findings)*				0.341
Agree	52 (55.9%)	41 (65.1%)	11 (36.7%)	
Disagree	28 (30.1%)	17 (27.0%)	11 (36.7%)	
Neutral	13 (14.0%)	5 (7.9%)	8 (26.6%)	
M (SD)	2.73 (1.24)	2.60 (1.10)	3.03 (1.12)	
*I want clinical guidelines and/or guidance on what to do with the RTPM information*				0.252
Agree	51 (54.8%)	39 (61.9%)	12 (40.0%)	
Disagree	24 (25.9%)	14 (22.2%)	10 (33.4%)	
Neutral	18 (19.3%)	10 (15.9%)	8 (26.6%)	
M (SD)	2.63 (1.10)	2.50 (1.09)	2.93 (1.08)	

* *p*-value of Chi-square or Fisher’s Exact Test (agree vs. disagree—neutral positioned according to question).

### 3.4. Other feedback

A total of 86 respondents provided an answer to the open-ended questions:

*What would make real time prescription monitoring tools more useful in clinical practice?*

*Any other comments?*

The most common theme focused on system quality and functionality of the log in process (30.2%) and the majority of the respondents were located in Queensland (65.0%). Practitioners stated that a streamlined log in process would make the tool easier to use.

In addition, participants described time barriers and disrupted workflow related to system infrastructure and design (20.9%) and requested tool integration with dispensing or prescribing software as a solution (13.9%). Comments included:

*“…need better infrastructure to reduce waiting/loading times…main barrier is being stuck at the “spinning wheel of doom” page…some days it doesn’t let me log in at all so therefore it is useless”* (medical practitioner, Queensland).

*“Real time monitoring has helped me improve my clinical judgement but takes a long time to access”* (pharmacist, Queensland).

*“It is very useful for information gathering at admission. But it is very burdensome for a hospital pharmacist at discharge because integration with hospital systems was considered out of scope. Disappointing”* (pharmacist, Victoria).

This was followed by the topic of mandated use that emerged as a point of interest for pharmacists who stated that RTPM tool use should be mandatory for all prescribers across all care settings (16.3%). Comments included:

*“…have looked at access history many times and saw only other pharmacists looking at it”* (pharmacist, Victoria).

*“The system is not useful when only a small percentage of providers are actively using it”* (pharmacist, New South Wales).

*“…make real time prescription monitoring mandatory for all doctors”* (pharmacist, Victoria).

*“There have to be stronger legal ramifications for practitioners not checking RTPM”* (pharmacist, Victoria).

Finally, respondents requested improved data standardization such as expanding the number of monitored medicines to include tramadol, pregabalin, benzodiazepines and anabolic steroids (9.3%) and improving the data presentation format to facilitate data interpretation as well as including more detailed data such as full patient medication history and indications for use, hospital prescribed and dispensed medication, and the daily dose dispensed (8.1%).

## 4. Discussion

### 4.1. Barriers and facilitators to RTPM tool use

The majority of participants reported that they had the necessary software and hardware to access the RTPM tool, however prescribers were more likely to report difficulties accessing a computer or the internet at their workplace compared with pharmacists. It is common knowledge that information technology has been part of the healthcare landscape for well over two decades and practitioners routinely use computers and the internet in practice. Previously, few studies reported on the availability of technology as a barrier to PDMP use including limited access to phones, computers, and the internet (14–16) and they did not discuss frequency. Harocopos et al. (19) discussed the association between technologically challenged older practitioners, those experiencing technology burnout and lack of PDMP use.

A significant proportion of participants reported that the RTPM information was easy to interpret. Furthermore, open-ended responses requested enhanced tool functionality via access to additional clinical information and the monitoring of more medications that would necessitate clinical interpretation. In contrast, previous studies revealed that practitioners had difficulty interpreting PDMP data due to the sub-optimal design and visual display of data, a lack of overview and context, and missing data (16–18, 22). The opposing responses are likely related to several factors including heterogeneity in tool features and design, and practitioner participation in RTPM tool training and use of resources to support the interpretation of RTPM information. Approximately one-third of respondents reported that the tool was easy to use and even fewer that it was easy to access. This was supported by responses to the open-ended question indicating that users found the log in process and loading times cumbersome. System slowness was reported in previous studies related not only to lag time in updates and the system timing out, but also to the inability to query the system in real time, an issue that does not exist with Australian RTPM tools (12, 13, 15, 23). It is argued that improving ease of access will increase the uptake of practitioner tool use, therefore, further research can investigate these barriers and how to minimize their impact on tool use.

Another perceived barrier was a lack of RTPM tool integration with other practice software and with the workflow process leading to disrupted workflow. Open-ended responses also highlighted the lack of integration with other software systems. Similarly, software integration issues and a lack of guidance on how to integrate the PDMP into workflow has been identified in previous studies (15, 24). Keller et al. (25) described how clinicians who applied systematized protocols were successful in implementing risk mitigation strategies.

Respondents were equally divided on whether they had sufficient time to use the tool. This is inconsistent with previous research where time constraints was reported as the main barrier to PDMP use (12, 15, 18, 22–25). Physicians in the study by Radomski et al. (12) stated that PDMP use was challenging because of limited time and competing demands, a sentiment echoed by pharmacists and specialists alike (13, 25). It is possible that fewer participants in this study reported time constraints than in previous studies due to geographical differences in healthcare systems including organizational processes, diversity in health information technology systems, and heterogeneity of prescription monitoring tools.

Overall, participants were in favor of mandated use. This was further supported by the open-ended responses where practitioners perceived mandated use was associated with tool usefulness and that there should be legal consequences for non-compliance. Many jurisdictions in both the United States and Australia have mandated use, and studies indicate that while mandates may or may not increase PDMP practitioner use, a reduction in high-risk opioid prescribing was observed (22, 26–28). However, uncertainty remains on the association between mandate laws and optimal PDMP engagement (26). Williams et al. (29) concluded that mandates were important from the context of public policy and were more effective than voluntary access. Radomski et al. (12) suggests that mandated use is necessary to improve patient outcomes. Further research on mandates and patient outcomes is needed.

### 4.2. Training and resources related to RTPM tool use

Overall, respondents reported that they have received sufficient training and had access to resources on how to use the tool. These findings suggest that participants gained sufficient knowledge to competently navigate the RTPM tool. However, more than half the practitioners agreed that they wanted training to guide clinical actions and clinical guidelines on what to do with the RTPM findings. Previous research reported training barriers including lack of knowledge on how to use the system and lack of access to training and inconsistent guidelines on PDMP use (15, 18). Conversely, training in the form of webinars and continued education to enhance PDMP knowledge was perceived as a facilitator (24, 30). Practitioners working in community settings and those with 1–10 years practice experience were predictors of requesting training to guide clinical actions in response to RTPM information. Previous PDMP-related research has not examined these associations. It is possible that community practice settings lack the clinical governance and protocols that guide clinical actions compared with hospital settings. And practitioners with fewer years in practice may have less experience managing patients at risk of misuse. RTPM tools support clinical decision making but do not offer guidance on how to manage complex patients. Practitioners must still use their professional judgment to provide patient-centered care. Clinicians have access to a range of Australian and international opioid management guidelines and patient care standards that recommend the use of PDMPs and RTPM tools however it is common knowledge that practitioners do not always utilize guidelines (4, 5). This highlights the need for tailored training and education that optimizes RTMP tool use to better support clinical decisions and facilitate the implementation of evidence-based clinical interventions. Future research can investigate how practitioners respond to the RTPM information and what guidance and training they need to navigate and support decision making and deliver best quality in practice management.

### 4.3. Strengths and limitations

This pilot study investigated the barriers and facilitators related to practitioner use of RTPM tools in Australia. This study had a small sample size, a not uncommon occurrence for surveys of health practitioners (18, 31) however the findings can be used to inform further research. A high proportion of participants were from Victoria. This study may be subject to selection bias with practitioners interested in RTPM tools more likely to participate.

## Data availability statement

The raw data supporting the conclusions of this article will be made available by the authors, without undue reservation.

## Author contributions

DH contributed to the design of the study, data collection, data analysis, and manuscript write up. CL and HK contributed to the design of the study, data analysis, and edited the manuscript. All authors contributed to the article and approved the submitted version.

## Conflict of interest

DH is an AHPRA registered pharmacist.

The remaining authors declare that the research was conducted in the absence of any commercial or financial relationships that could be construed as a potential conflict of interest.

## Publisher’s note

All claims expressed in this article are solely those of the authors and do not necessarily represent those of their affiliated organizations, or those of the publisher, the editors and the reviewers. Any product that may be evaluated in this article, or claim that may be made by its manufacturer, is not guaranteed or endorsed by the publisher.
