# Supplementary material for: Barriers and facilitators related to healthcare practitioner use of real time prescription monitoring tools in Australia
Source: Front Public Health. 2023 Sep 12;11:1175791. doi: 10.3389/fpubh.2023.1175791 (PMC10522831; doi:10.3389/fpubh.2023.1175791)
Supplement: Supplementary file 1 [file Table_3.docx]

**Supplementary: Survey Instrument questions**

| ***Participant Information/Demographics*** | | |
| --- | --- | --- |
| **Description** | **Response** | **Field Type** |
| Age |  | Free text |
| Gender | Male/female/non-binary/prefer not to answer/I use a different term (please specify) | Multiple choice drop-down (single answer) |
| Healthcare practitioner type | Dental practitioner/medical practitioner/midwife/nurse practitioner/pharmacist/podiatrist | Multiple choice drop-down (single answer) |
| Practice setting | Community/emergency department/general practice/hospital/pain clinic/private clinic/other (please specify) | Multiple choice drop-down (single answer) |
| Main speciality | Community pharmacist/accredited pharmacist/advanced practice nurse/anaesthesiology/dentistry/emergency medicine/general medicine/obstetrics-gynaecology/oncology/paediatrics/pain medicine/primary healthcare/psychiatry/radiology/surgical/other (please specify) | Multiple choice drop-down (single answer) |
| Main practice state/territory | ACT/New South Wales/Queensland/South Australia/Tasmania/Victoria | Multiple choice drop-down (single answer) |
| Main practice area | Metro/rural/regional/remote | Multiple choice radio buttons (single answer) |
| Years in practice | <1 year/1-10 years/11-20 years/> 20 years | Multiple choice radio buttons (single answer) |
| Main practice hours  (based on an average week) | <10 hours/11-20 hours/>20 hours | Multiple choice radio buttons (single answer) |

| ***Real time prescription monitoring tool registration and use*** | | |
| --- | --- | --- |
| **Description** | **Response** | **Field Type** |
| Are you registered to use your state/territory real time prescription monitoring tool? | Yes/No | Yes/No |
| Have you ever used the real time prescription monitoring tool? | Yes/No | Yes/No |

| **Description** | **Response** | **Field Type** |
| --- | --- | --- |
| I am not registered because: | I don’t have time to register  I don’t know how to register  I did not know I had to register  I don’t want to use the real time prescription monitoring tool  It is not relevant to my practice or workplace  I do not have access to the real time prescription monitoring tool at my practice or workplace  I do not prescribe or dispense real time prescription monitored medicines  It is not mandatory in my state/territory  Other (please specify) | Checkboxes (multiple answers) |
| I have not used the real time prescription monitoring tool because: | I don’t have the time to use it  I don’t know how to use it  I did not know I had to use it  It is not relevant to my practice or workplace  I do not have access to the real time prescription monitoring tool at my practice or workplace  I do not prescribe or dispense real time prescription monitored medicines  It is not mandatory in my state/territory  Other (please specify) | Checkboxes (multiple answers) |

| ***Real time prescription monitoring tool use*** | | |
| --- | --- | --- |
| **Description** | **Response** | **Field Type** |
| How many times do you use the real time prescription monitoring tool (on a typical day)? | <1 time per day  1-2 times per day  3-5 times per day  >5 times per day | Multiple choice radio buttons (single answer) |
| Real time prescription monitoring tool information is useful | Strongly agree  Agree  Neutral  Disagree  Strongly disagree | Multiple choice radio buttons (single answer)  *5 point Likert* |
| Real time prescription monitoring is a valuable tool for informing clinical decision making | Strongly agree  Agree  Neutral  Disagree  Strongly disagree | Multiple choice radio buttons (single answer)  *5 point Likert* |
| Real time prescription monitoring is considered a priority tool in my workplace | Strongly agree  Agree  Neutral  Disagree  Strongly disagree | Multiple choice radio buttons (single answer)  *5 point Likert* |
| I use the real time prescription monitoring tool to: | Check patient medication history before I prescribe/dispense real time prescription monitored medicines  Check that the patient is not attending multiple doctors/and or multiple pharmacies  Check for evidence of possible misuse or abuse (eg early refill, dose escalation)  Check for concomitant use of medicines and/or drug interactions  Monitor prescription refills (compliance) or medication use  Improve communication with patient to discuss use of real time prescription monitored medicines  Confirm patient story/presentation  Other (please specify) | Checkboxes (multiple answers) |
| I use the real time prescription monitoring tool for: | All patients/new patients only/all opioid prescribing-dispensing only/all real time prescription monitored medicines/when I have a clinical intuition/other (please specify) | Checkboxes (multiple answers) |

| ***Healthcare practitioner clinical practice perspective of real time prescription monitoring tool*** | | |
| --- | --- | --- |
| **Description** | **Response** | **Field Type** |
| Real time prescription monitoring improves patient care/outcomes | Strongly agree  Agree  Neutral  Disagree  Strongly disagree | Single answer radio buttons  *5 point Likert* |
| Real time prescription monitoring improves clinical practice |  |  |
| Real time prescription monitoring optimises medicine use |  |  |
| Real time prescription monitoring improves co-ordination of patient care |  |  |
| Real time prescription monitoring reduces the risk of potential harm (eg overdose) |  |  |
| I have changed my prescribing/dispensing practices as a result of real time prescription monitoring information |  |  |
| I have changed my clinical decisions as a result of real time prescription monitoring information |  |  |
| Practitioner judgment is better than real time prescription monitoring |  |  |
| Real time prescription monitoring inhibits the ability to prescribe or dispense |  |  |

| ***Real time prescription monitoring tools in clinical practice (medical practitioners)*** | | |
| --- | --- | --- |
| **Description** | **Response** | **Field Type** |
| As a result of real time prescription monitoring information, I have: | Continued to prescribe  Refused to supply a prescription for a real time prescription monitored medicine  Prescribed/organised staged supply  Prescribed smaller quantities  Tapered dose(s)  Rotated opioid(s)  Prescribed an alternative non-opioid medicine  Prescribed naloxone  Offered non-pharmacological strategies (eg exercise, hydrotherapy)  Arranged a contract or agreement with the patient  Ordered a urine drug screen/toxicology  Referred the patient to pain management specialist/service  Referred the patient to mental health specialist/service  Referred the patient for substance use disorder/addiction management services (not opiate replacement/medication-assisted treatment of opioid dependence program/service)  Prescribed opiate replacement therapy/medication-assisted treatment of opioid dependence or referred patient to an opiate replacement/medication-assisted treatment of opioid dependence program/service  Contacted the dispensing pharmacy/pharmacies to discuss real time prescription monitoring information  Contacted other prescribers to discuss real time prescription monitoring information  Developed a management plan with the patient  Provided the patient with written information or other resources  Discussed the real time prescription monitoring information with the patient  Counselled/discussed with the patient the risks of misuse/abuse/safety concerns  Arranged a medication management review  Discharged the patient from the practice  Reported the patient to law enforcement  Nothing (ignored the real time prescription monitoring information)  Other (please specify) | Checkboxes (multiple answers) |

| ***Real time prescription monitoring tools in clinical practice (pharmacists)*** | | |
| --- | --- | --- |
| **Description** | **Response** | **Field Type** |
| As a result of real time prescription monitoring information, I have: | Continued to dispense  Refused to dispense a prescription for a real time prescription monitored medicine  Organised or offered staged supply  Dispensed a smaller quantity  Recommended an alternative non-opioid medicine  Referred the patient back to their prescriber  Offered naloxone  Encouraged return of unused medicines to the pharmacy  Offered non-pharmacological strategies (eg exercise, hydrotherapy)  Arranged a contract or agreement with the patient  Referred the patient to pain management specialist/service  Referred the patient to mental health specialist/service  Referred the patient for substance use disorder/addiction management services (not opiate replacement/medication-assisted treatment of opioid dependence program/service)  Discussed opiate replacement therapy/medication-assisted treatment of opioid dependence or referred patient to an opiate replacement/medication-assisted treatment of opioid dependence program/service  Contacted the dispensing pharmacy/pharmacies to discuss real time prescription monitoring information  Contacted the prescriber(s) to discuss real time prescription monitoring information  Developed a management plan with the patient  Provided written information and/or resources to the patient  Discussed real time prescription monitoring findings with the patient  Counselled/discussed with the patient the risks of misuse/abuse/safety concerns  Arranged or conducted a medication management review  Discharged the patient from the pharmacy practice  Reported the patient to law enforcement  Nothing (ignored the real time prescription monitoring information)  Other (please specify) | Checkboxes (multiple answers) |

| ***Barriers and facilitators related to the use of real time prescription monitoring tools*** | | |
| --- | --- | --- |
| **Description** | **Response** | **Field Type** |
| I have limited or no access to a computer or the internet at my workplace | Strongly agree  Agree  Neutral  Disagree  Strongly disagree | Single answer radio buttons  *5 point Likert* |
| I have limited or no access to a real time prescription monitoring tool at my workplace |  |  |
| The real time prescription monitoring tool is easy to use |  |  |
| The real time prescription monitoring tool is easy to access |  |  |
| I have sufficient time to use the real time prescription monitoring tool |  |  |
| The real time prescription monitoring tool is integrated in my workflow process |  |  |
| The real time prescription monitoring tool does not disrupt workflow |  |  |
| Real time prescription monitoring data is easy to interpret |  |  |
| My workplace has a policy to support real time prescription monitoring tool use |  |  |
| I am confident in my response to suspected abuse or misuse |  |  |
| I have sufficient resources to act on real time prescription monitoring information |  |  |
| I am concerned about the legal ramifications of using the real time prescription monitoring tool |  |  |
| I am concerned about patient privacy or data security when using the real time prescription monitoring tool |  |  |
| Real time prescription monitoring tool use should be voluntary |  |  |
| Real time prescription monitoring tool use takes time away from patient care |  |  |
| Real time prescription monitoring tool use should be reimbursed |  |  |

| ***Real time prescription monitoring tools training and resources*** | | |
| --- | --- | --- |
| **Description** | **Response** | **Field Type** |
| I received sufficient training on how to use the real time prescription monitoring tool | Strongly agree  Agree  Neutral  Disagree  Strongly disagree | Single answer radio buttons  *5 point Likert* |
| I have access to sufficient resources on how to use the real time prescription monitoring tool |  |  |
| I have access to sufficient real time prescription monitoring tool support (technology, peer, practice support) |  |  |
| I want training on how to interpret real time prescription monitoring tool data |  |  |
| I want training to guide actions I can take (as a result of real time prescription monitoring findings) |  |  |
| I want clinical guidelines and/or guidance on what to do with the real time prescription monitoring information |  |  |

| ***Further feedback*** | |
| --- | --- |
| **Description** | **Field Type** |
| What would make real time prescription monitoring tools more useful in clinical practice? | Free text |
| Any other comments? | Free text |
